# Multi-PLI: interpretable multi‐task deep learning model for unifying protein–ligand interaction datasets

**DOI:** 10.1186/s13321-021-00510-6

**Published:** 2021-04-15

**Authors:** Fan Hu, Jiaxin Jiang, Dongqi Wang, Muchun Zhu, Peng Yin

**Affiliations:** Guangdong-Hong Kong-Macao Joint Laboratory of Human-Machine Intelligence-Synergy Systems, Shenzhen Institutes of Advanced Technology, Chinese Academy of Sciences, Shenzhen, 518055 China

**Keywords:** Interpretable, Deep learning, Multi‐task, Drug discovery

## Abstract

The assessment of protein–ligand interactions is critical at early stage of drug discovery. Computational approaches for efficiently predicting such interactions facilitate drug development. Recently, methods based on deep learning, including structure- and sequence-based models, have achieved impressive performance on several different datasets. However, their application still suffers from a generalizability issue because of insufficient data, especially for structure based models, as well as a heterogeneity problem because of different label measurements and varying proteins across datasets. Here, we present an interpretable multi-task model to evaluate protein–ligand interaction (Multi-PLI). The model can run classification (binding or not) and regression (binding affinity) tasks concurrently by unifying different datasets. The model outperforms traditional docking and machine learning on both binary classification and regression tasks and achieves competitive results compared with some structure-based deep learning methods, even with the same training set size. Furthermore, combined with the proposed occlusion algorithm, the model can predict the important amino acids of proteins that are crucial for binding, thus providing a biological interpretation.

**Supplementary Information:**

The online version contains supplementary material available at 10.1186/s13321-021-00510-6.

## Introduction

The development and approval of a new drug takes more than 10 years and costs almost 2 billion dollars. Identification of the interactions between proteins and ligands are critical at early stage of the drug discovery process. Computational methods for identifying possible ligands to target proteins at the initial phase of drug discovery indeed reduce the cost and improve the success rates of new drug development [1, 2]. However, traditional methods have limitations, for example, the dependence on expert knowledge may lead to low efficiency in screening and the limited results. Specifically, these conventional structure-based methods need to first simulate the binding poses of proteins and ligands and then calculate their binding energies, which tends to be restricting the computational efficiency and accuracy. In recent years, researchers in this field have paid more attention on machine learning based methods [3, 4]. However, the fundamental limitation of models such as support vector machine is that they still rely on expert knowledge-based manual feature engineering.

Recently, deep learning, which refers to an algorithm for numerous layers of nonlinear transformations, has achieved great success in many fields [5–7]. One main advantage of is that deep learning algorithm learns and extracts information from raw data without manual feature extraction. Inspired by the remarkable success, many researchers have applied deep learning into the field of drug discovery [8–14]. Wallach et al. proposed a method which based on convolutional neural network (CNN), an algorithm of deep learning, to divide active and inactive compounds for a given protein [9]. In their study, their model outperformed other traditional methods on the Directory of Useful Decoys Enhanced (DUD-E) benchmark. In another study, Ragoza et al. described a CNN-based scoring function using a comprehensive 3-dimensional (3D) representation of a protein-ligand complex as input. They showed a better performance on virtual screening and pose prediction than the classical docking method AutoDock Vina [10]. Similarly, Stepniewska-Dziubinska et al. introduced a model taking a 3D grid representation structure as input and processing it by CNN. Rather than simply identifying whether the ligand can bind to the target, their model can accurately predict the binding affinity of the protein–ligand complex [11]. It should be noted that methods taking the 3D structure of a protein-compound complex as input, which is similar to traditional docking, may also be disadvantaged by the lack of data, especially for targets without structural information. Therefore, several studies have introduced methods that use only 1D sequences as input. Wan et al. applied the “word embedding” algorithm, which is widely used in natural language processing, to process raw protein and compound data into two separate compressed vectors [15]. Then, the two embedding vectors were fed into a deep neural network to predict the binding possibility. Similarly, to predict the binding value, Öztürk et al. proposed a model known as DeepDTA that applies convolution operations to protein and drug sequences separately, and their model obtained better results than other methods on kinase datasets [12]. Considering the model interpretability, Lee et al. performed convolution on various lengths of amino acid subsequences to capture local residue patterns [14]. They pooled the maximum convolution results from each filter to highlight important regions for prediction, and thus provided a partial explanation of their model. However, the robustness and applicability of a model are limited if the model is restricted to only one identical dataset or single task, namely, either classification or regression.

**Fig. 1:**
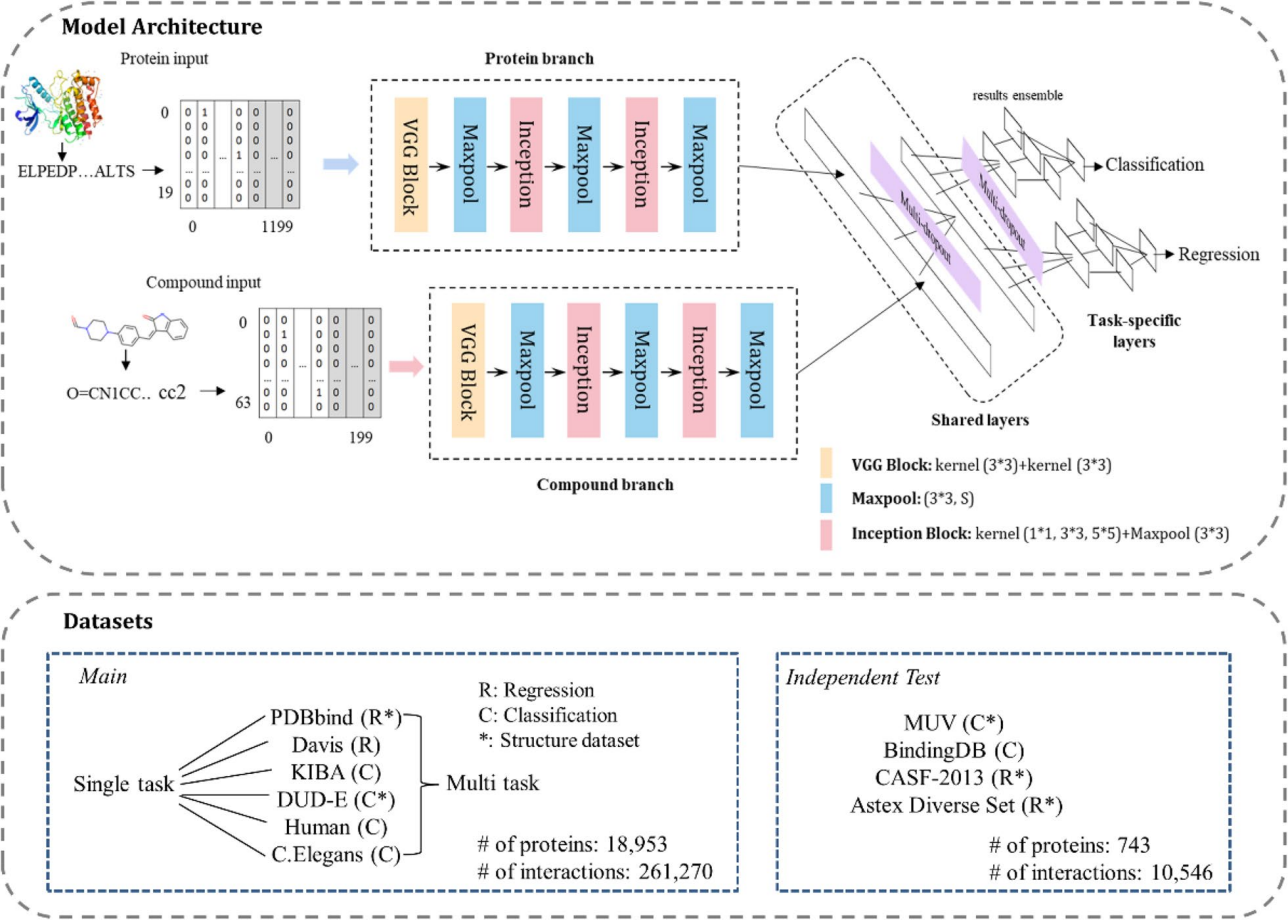
Schematic overview of our method. The proposed model consists of two parts: protein/ligand feature extraction from sequence/SMILES and interaction prediction by shared and task-specific layers. The tasks are defined as: binary classification (protein-ligand binding or not) and regression (protein-ligand binding affinity). The main datasets consist of two regression sets and four classification sets, in which PDBbind and DUD-E have structural data. Four independent sets are used to test the generalizability of the model

Inspired by previous studies, here we present an interpretable multi-task model to evaluate protein-ligand interactions. Using sequence data, the model can run classification task (binding or not) and regression task (binding affinity) concurrently, based on corresponding labels. As shown in Fig. 1, the CNN blocks consist of VGG, Inception and Maxpool modules, which are used to extract latent features from raw sequence/SMILES of proteins/compounds, and then fully connected layers are employed to process the combined vectors. The key idea is to perform multiple convolutions operations with multiple kernels (i.e., 1 × 1, 3 × 3 and 5 × 5 convolutional layers) and pooling layers (i.e., 3 × 3 max pooling layer) simultaneously in parallel within the same layer. To avoid overfitting, a multi-dropout layer is added after each dense layer, where each multi-dropout layer consists of five units that generate random dropout values, and then the final dropout is calculated by the weighted mean of these values to achieve better performance. Similarly, the ensemble result refers to the final output composed of the five different values generated by the last dense layer. In general, this parallel algorithm decreases the variance/bias and thus leads to a more efficient model performance. A total of 261,270 interactions from six main datasets, including two regression sets (PDBbind and Davis) and four classification sets (DUD-E, *Human*, *C. elegans* and KIBA), are used in this study, as well as 10,546 interactions from three independent sets: CASF-2013, Astex Diverse, MUV and BindingDB.

The proposed model outperforms traditional docking and machine learning on both binary classification and regression tasks and achieves competitive results compared with some structure-based deep learning methods, even with the same training set size. Additionally, the model can be used to identify the important sites of the input data in combination with the designed occlusion algorithm, thus providing a biological interpretation.

## Results and discussion

### Performance of single-task model

**Fig. 2:**
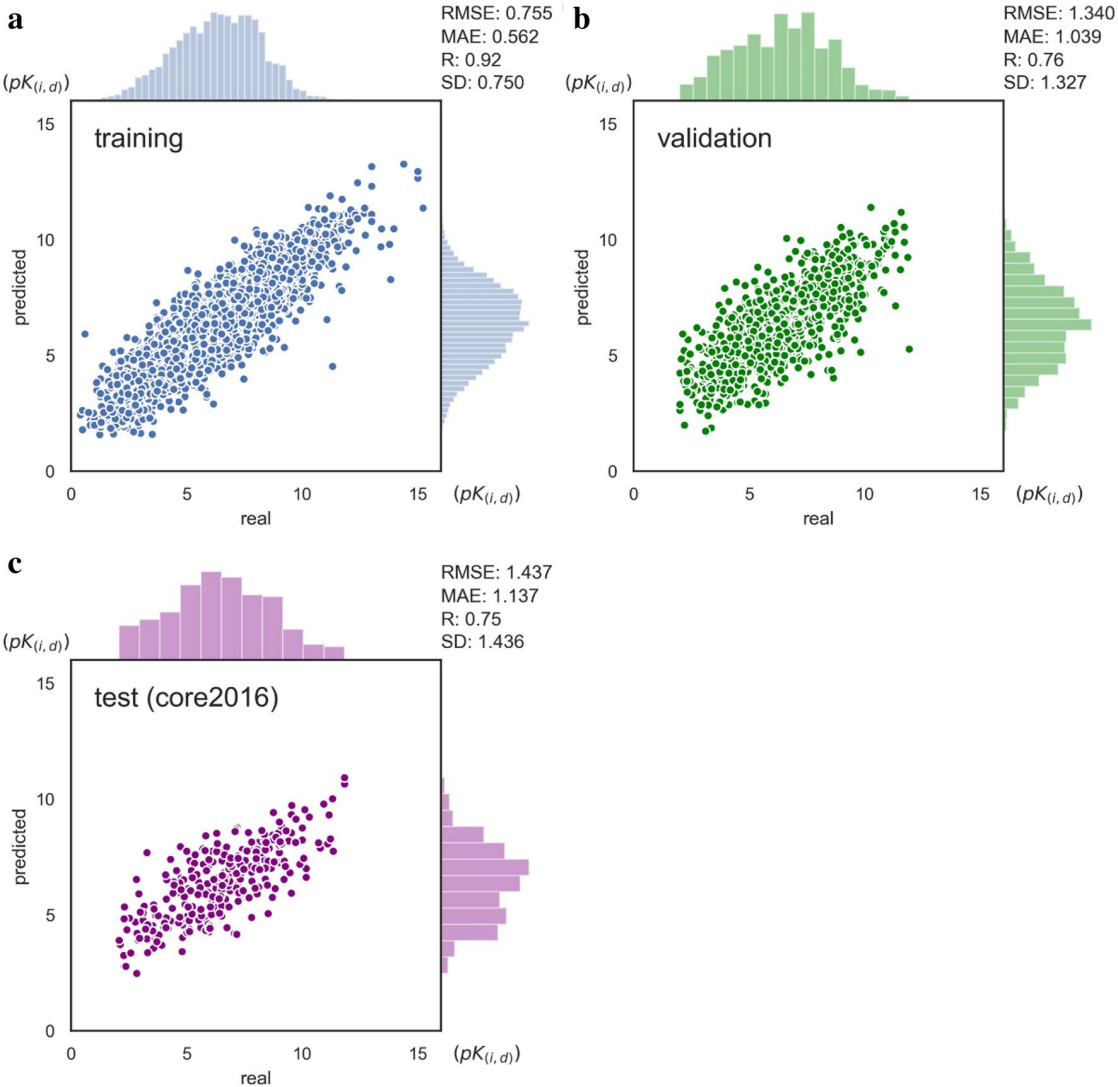
Model performance on PDBbind (regression). **a** Training set, RMSE = 0.75, R = 0.92; **b** validation set, RMSE = 1.34, R = 0.76; **c** test set (core2016), RMSE = 1.437, R = 0.75. Coordinates of x and y: *pK*(_*i,d*_) (−logK_i_ or −logK_d_). Histogram: affinity distributions of real (x) and predicted (y) samples (*pK(*_*i,d*_*)*)

We first train and evaluate the model with a single task on the PDBbind dataset. A single task means that model is trained and evaluated on each individual dataset for either regression or classification. PDBbind v.2016 is split the same as Pafnucy [11], with the whole core set (core2016) as the external test set. Figure 2 displays the predicted values against the real binding values of the protein-ligand complex on PDBbind. RMSE and Pearson’s correlation coefficient R are used to calculate the differences and the linear correlation, respectively, between the predicted and real values. As shown, our model achieves the lowest error on the training set with RMSE = 0.75, R = 0.92 and performs well on the validation set with RMSE = 1.34, R = 0.76 and on the test set (core2016) with RMSE = 1.437, R = 0.75. These results show that our model performs well on both the validation and the test sets.

**Fig. 3:**
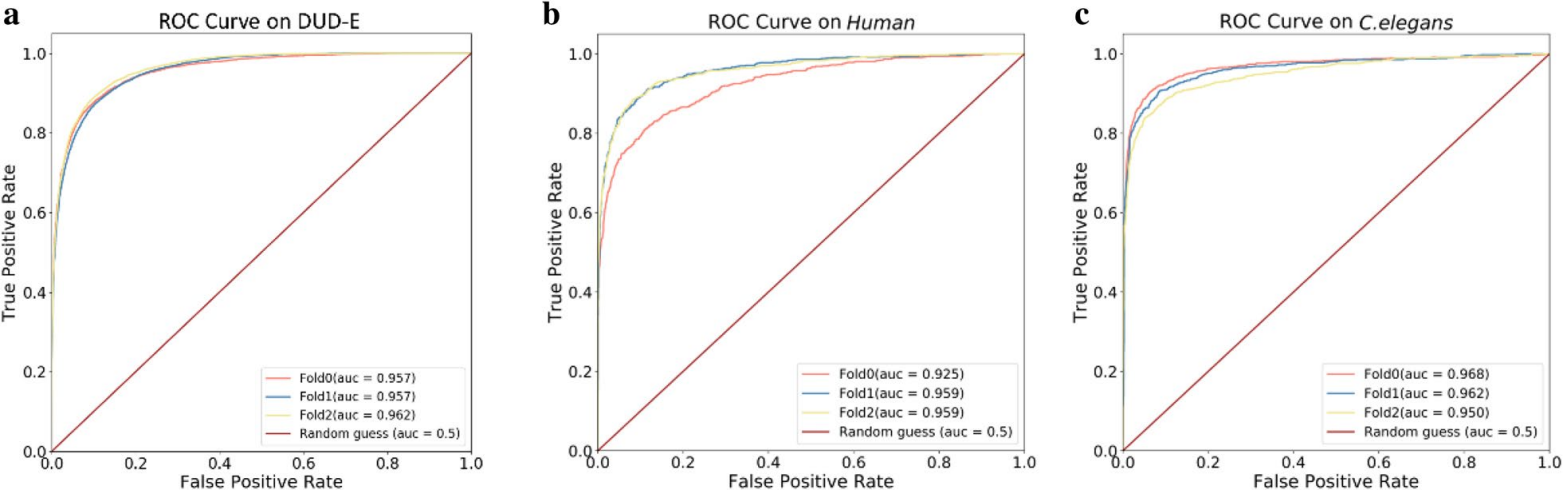
Model performance on DUD-E, *Human* and *C. elegans* (classification). Three-fold cross-validation and random-guess ROC curves plotted in different colors. **a** DUD-E, mean AUC = 0.959; **b** Human, mean AUC = 0.948; **c** *C. elegans*, mean AUC = 0.960

We use 3-fold cross-validation to evaluate the model performance on three classification datasets, namely, DUD-E, *Human* and *C. elegans*, as shown in Fig. 3. The data from *Human* and *C. elegans* are randomly split as in previous studies [13, 16]. In contrast, several recent studies reported that the bias inherent in DUD-E may lead to perfect AUC results for any machine learning method if using randomly split intra-targets [17, 18]. Thus, the DUD-E data are split according to 102 protein clusters using global sequence alignment [19] to ensure that targets with greater than 80% sequence identity are included in the same fold during cross-validation, so that no ligand-protein information for a target in the test set is included in the training set. The negative-to-positive ratios of are set to 3:1 for all three datasets. The AUC results show that our model performs well on the *Human* and *C. elegans* datasets with mean AUCs of 0.948 and 0.960, respectively, and on DUD-E with a mean AUC = 0.959 even when target clustering is applied (Fig. 3).

Recent studies showed that SMILES-based methods can model different biological activity and physicochemical properties [20]. Therefore, we have performed an ablation study on these datasets to explore the importance of each protein/ligand part in our model. For DUD-E, ligand only model has achieved similar result to complete model whereas protein only model does not work at all, which suggests the ligand part plays the decisive role on DUD-E dataset (Additional file 1: Table S1). But for *Human* and *C. elegans*, the complete model (i.e., with both protein and ligand parts) achieves the best performance. This result has also been observed on PDBbind (Additional file 1: Table S2). Next, we count the numbers of unique protein and ligand (after removing duplicates by sequence/smiles) on each dataset. DUD-E has the extremely imbalanced ratio of unique ligands to proteins (88,092 ligands and 102 proteins) whereas other datasets have more balanced ligand to protein ratios. Taken together, these results suggest that ligand only model could achieve competitive result to protein/ligand model on dataset where ligand dominates. Whereas the protein/ligand model would achieve better performance than protein or ligand only models on relative balanced dataset.

### Unification of different datasets

One limitation of deep learning-based drug discovery is the lack of data, which may partially be attributed to the heterogeneity of different datasets. For example, some datasets have accurate protein-ligand binding values, while other datasets provide positive and negative ligands for targets, as exhibited in the last section. In addition, different datasets may have their own protein-ligand interaction space, and models trained on a single dataset do not generalize well to other datasets. For example, DeepDTA optimized on kinase datasets achieved poorer performance on an independent set than did DeepConv-DTI, which was trained on diverse data [14]. Moreover, this limitation creates obstacles when comparing different models. Although the loss function could be redefined for different task comparisons, the model parameters will not work well on feature extraction and prediction tasks, and thus, the model actually needs to be retrained. Given these facts, we propose a multi-task framework to unify datasets with different labels.

**Fig. 4:**
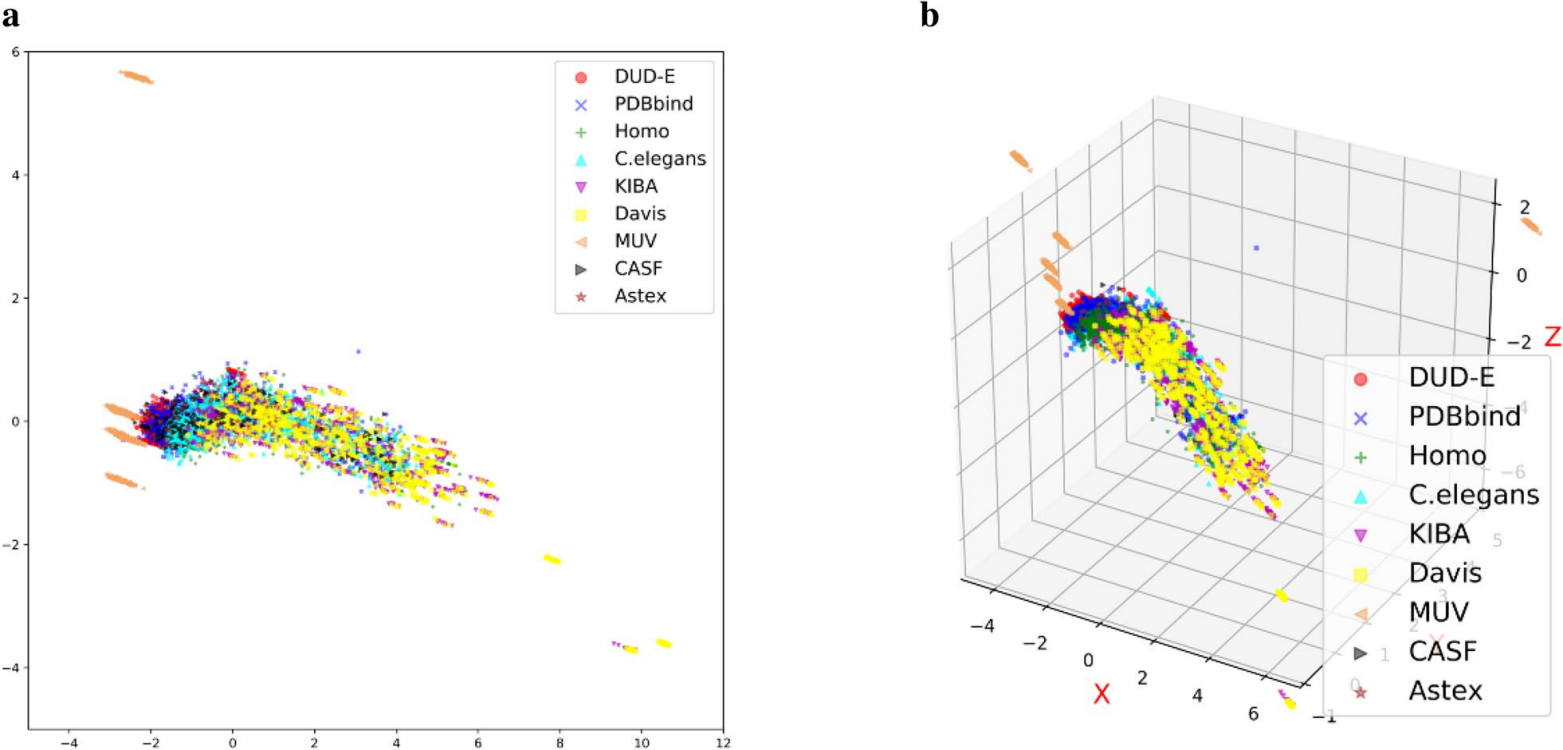
PCA analysis of all datasets used in this study. **a** PC1 and PC2; **b** PC1, PC2 and PC3. Randomly samples from each dataset are compared after PCA reduction. Main datasets: DUD-E (red), PDBbind (blue), Human (green), C. elegans (cyan), KIBA (purple) and Davis (yellow). Independent test sets: MUV (peachpuff), CASF2013 (gray), Astex Diverse (peru)

An analysis of the used protein–ligand datasets, including the independent sets, is given here. One thousand samples per dataset are selected randomly, and one hot protein and ligand encodings are concatenated for each sample. Then, these selected protein-ligand pairs are compared across the datasets after applying a dimensionality reduction method, principal component analysis (PCA). The distributions of the nine datasets in both the 2D and 3D PC spaces are visualized in Fig. 4a, b. Although these datasets are distributed close together in space, slight differences can be seen. DUDE (red) is distributed similar to PDBbind (blue), note that both DUD-E and PDBbind are structure-based datasets. KIBA (purple) and Davis (yellow) are distributed closer than other pairs of datasets, as are *Human* (green) and *C. elegans* (cyan). It makes sense because KIBA and Davis are both kinase sets while *Human* and *C. elegans* were built by the same pipeline [16]. These results indicate that the six main datasets differ in characterization, and it is possible that a broader distribution space adequately covered by more samples could make the model more robust and practical. Regarding the independent sets, CASF and Astex Diverse are close to the main datasets in the space, whereas MUV is quite far from the other sets. These distributions may lead to good prediction results on CASF and Astex Diverse but a relatively poor performance on MUV.

### Performance comparison with other methods

The multi-task model is trained once on the main datasets. During training, the loss changes are monitored. The model is saved on one epoch as final model if the loss of validation set starts to increase while the loss of training set still reducing. For the multi-task model, the final epoch for classification and regression is 9 and 20, respectively, which suggests that the regression task is a bit harder to cover than the classification task. Then, we compare our single- and multi-task models with other methods on the five main datasets and four independent sets. To facilitate fair comparisons, the test sets are similar for all methods on the given datasets. It should be noted that the sequences of proteins with structural information are extracted from their structural data (e.g., proteins from PDBbind and DUD-E), whereas the sequences of proteins from sequence-only datasets are obtained from the original data sources (e.g., *Human* and *C. elegans*).

On PDBbind, a benchmark dataset for structure-based methods, we use exactly the same training/validation/test sets of Pafnucy [11], a structure-based deep learning model that performs excellently on PDBbind. Pafnucy achieves RMSE = 1.44, R = 0.72 and RMSE = 1.42, R = 0.78 on the validation and test (core2016) sets, respectively, while our single-task model exhibits RMSE = 1.34, R = 0.76 on the validation set and RMSE = 1.44, R = 0.75 on the test (core2016) set (Fig. 2b, c), and the multi-task model achieves RMSE = 1.18, R = 0.63 (a combination of the PDBbind and Davis validation sets) and RMSE = 1.49, R = 0.74 on the test (core2016) set, respectively (Table 1). These results suggest that the single- and multi-task models both perform slightly better on the validation set but slightly worse on the test (core2016) set compared to Pafnucy.

**Table 1:** Performance comparisons on PDBbind and the modified Davis sets

Dataset	Method	RMSE	R	SD
PDBbind (core2016)	SVM	1.77	0.48	1.68
	DeepDTA	1.51	0.61	1.50
	Pafnucy	**1.42**	**0.78**	**1.37**
	Single-task on PDBbind	1.44	0.75	1.44
	Single-task on Davis	2.51	0.32	2.06
	Multi-task	1.49	0.74	1.46
Davis	SVM	0.98	0.36	0.96
	DeepDTA	0.95	0.43	0.94
	Single-task on Davis	**0.92**	**0.46**	**0.92**
	Single-task on PDBbind	1.26	0.03	1.06
	Multi-task	0.99	0.38	0.97

The result of Pafnucy on PDBbind is derived from [11]

Best values are higlhlighted in bold

We compare the performance of several approaches on the Davis dataset. As mentioned, Davis is a kinase dataset without structural information for protein-ligand interactions. To avoid an imbalanced data distribution for our multi-task model, the samples with pK_d_ = 5, which account for the majority of the dataset, are excluded. As shown, our single-task model outperforms SVM and DeepDTA on the modified Davis dataset, while the multi-task model achieves similar results. It should be noted that our proposed model has a similar structure to DeepDTA, but we use several inception blocks during protein/ligand feature extraction to capture different sizes of features and combine them together. Additionally, a multi-dropout layer is added between each pair of dense layers to reduce overfitting of the model, while the ensemble results decrease variance/bias and lead to a more efficient model performance. Not surprisingly, the single-task trained on the Davis set achieves terrible results on PDBbind with RMSE = 2.51, R = 0.32, and the single-task model trained on PDBbind performs poorly on Davis with RMSE = 1.26, R = 0.03. These results suggest that although the multi-task model may result in a small performance loss on individual datasets, the model applicability is increased effectively by unifying multiple datasets.

**Table 2:** Performance comparisons on the independent CASF-2013 and Astex Diverse sets

Independent set	Method	RMSE	R	SD
CASF-2013	X-Score	–	0.61	1.78
	Pafnucy	**1.62**	**0.70**	**1.61**
	Single-task on PDBbind	1.64	0.68	1.65
	Multi-task	1.80	0.62	1.79
Astex Diverse	X-Score	1.55	0.52	1.48
	Pafnucy	1.43	0.57	1.43
	Single-task on PDBbind	**1.38**	**0.64**	**1.33**
	Multi-task	1.43	0.61	1.37

The results of X-Score and Pafnucy are derived from [11, 21]

Best values are higlhlighted in bold

To evaluate the generalizability of the model, we also compare the methods on two independent sets. As shown in Table 2, the single- and multi-task models both achieve better results on the Astex Diverse set with RMSE = 1.38, R = 0.64 and RMSE = 1.43, R = 0.61, respectively, whereas Pafnucy obtains RMSE = 1.43, R = 0.57. On CASF-2013, the single-task model achieves good performance with RMSE = 1.64, R = 0.68 compared to Pafnucy with RMSE = 1.62, R = 0.70. In contrast, the multi-task model performs worse with RMSE = 1.80, R = 0.62. It should be emphasized that our model uses only 1D sequences/SMILES of proteins/ligands to achieve competitive results compared to structure-based deep learning models. The applicability of structure-based models is often restricted by the lack of structural data. For example, Davis dataset do not have structural information. Therefore, Pafnucy cannot be employed or compared on this dataset. In contrast, our multi-task model can be used and compared on multiple datasets regardless of the types of data or labels in the dataset.

For another structural benchmark dataset, DUD-E, we compare our models with AutoDock Vina (a docking scoring function) [22], AtomNet [9], 3D-CNN (a structure-based deep learning model) [10] and CNN/Graph [13]. Here, the DUD-E dataset is split into a training set (61 targets), a validation set (21 targets), and a test set (20 targets) by target clustering, which is similar to AtomNet and CNN/Graph (both of which split into 72 training targets and 30 testing targets). Our single-task and multi-task models achieve AUC values of 0.973 and 0.971, respectively, on the test set (Table 3), thereby outperforming the other methods, including Vina with AUC = 0.716, 3D-CNN with AUC = 0.868, AtomNet with AUC = 0.895 and CNN/Graph with AUC = 0.950, when similar target clustering is used.

**Table 3:** Performance comparison on the DUD-E dataset

Metric	Vina	AtomNet	3D-CNN	CNN/Graph	Single-task on DUD-E	Multi-task
AUC	0.716	0.895	0.868	0.950	**0.973**	0.971

The AUC scores of Vina, 3D-CNN, AtomNet and CNN/Graph are derived from [9, 10, 13]

Best values are higlhlighted in bold

We also compare methods on another independent set, MUV. As shown in Table 4, MUV is a very challenging set in which all methods achieve low AUCs, which is consistent with previous findings [17, 23]. Among these results, the deep learning-based methods, including our single- and multi-task models, perform poorer than Vina with an average AUC = 0.549 for all targets. The results for each target are listed in Additional file 1: Table S4. It is probably difficult for a complex deep learning method to predict samples that are greatly different from the training set from which it has learned features. As depicted in Fig. 4, MUV is far from the other datasets in the interaction space. This may further support that divergence between the distributions of the training and test sets would lead to biased predictions.

**Table 4:** Performance comparison on the independent MUV dataset

Metric	Vina	3D-CNN on DUD-E	3D-CNN on DUD-E/CASR	Single-task on DUD-E	Multi-task
AUC	**0.549**	0.522	0.499	0.453	0.443

The AUC scores of Vina and 3D-CNN are derived from [10]

Best values are higlhlighted in bold

For the *Human* and *C. elegans* datasets, a graph-based method [13] provided state-of-the-art results with AUC = 0.950 on the *Human* set and AUC = 0.971 on the *C. elegans* set with a negative-to-positive ratio = 3. For comparison, with a negative-to-positive ratio = 3, our single- and multi-task models achieve better results with AUC = 0.958 and 0.961, respectively, on *Human*, and competitive results with AUC = 0.963 and 0.970 on *C. elegans* (Table 5). Moreover, a more detailed evaluation about model is listed in the Additional file 1: Table S3, the results show that multi-task model achieves better performance than single-task model on most evaluation metrics including balanced accuracy, recall, F1 score and MCC.

**Table 5:** Performance comparison on the *Human* and *C. elegans* sets

Dataset	Metric	k-NN	SVM	CNN/Graph	Single-task	Multi-task
*Human*	AUC	0.904	0.942	0.950	0.958	**0.961**
* C. elegans*	AUC	0.892	0.901	0.971	0.963	**0.970**

The AUC scores of k-NN, SVM, CNN/Graph are derived from [13]

Best values are higlhlighted in bold

To further verify the applicability of our multi-task model, we have compared the performance of single-task models and multi-task model on an independent classification test set derived from BindindDB [24]. For the single-task on regression sets (Davis and PDBbind), we use the *pK*(_*i,d*_) value of 6 (IC_50_ = 1µM) as threshold to divide positive and negative. For example, if the predicted value of a sample is 6.5 then it is regarded as positive. It should be noted that the single-tasks for regression output a binding affinity ranging from 0 to 15, rather than a probability value of 0 to 1. Therefore, the corresponding AUC cannot be calculated. As shown in Table 6, the single-task models trained on each single classification dataset achieve worse results on this independent set than the multi-task model which probably has benefited from the regression task, as suggested by the good result achieved by the single-task on PDBbind. This result suggests a better applicability of the multi-task model by leveraging various data with different labels.

**Table 6:** Performance comparison of our models on the independent BindingDB set

Metric	Single-task on DUD-E	Single-task on Human	Single-task on *C.elegans*	Single-task on KIBA	Single-task on Davis	Single-task on PDBbind	Multi-task
Accuracy	0.521	0.534	0.532	0.480	0.532	0.587	**0.580**
AUC	0.501	0.512	0.547	0.432	–	–	**0.613**

Best values are higlhlighted in bold

Combining the above results, the multi-task model seems to perform better than the single-task model on classification tasks but slightly worse on regression tasks. This can be ascribed to some possible explanations. First, the two regression datasets used in this study have different distributions of binding values. The PDBbind set fits well to a normal distribution whereas Davis obeys a skewed distribution distributed mostly at 6 (Additional file 1: Figure S1). The two merged regression datasets fit the multi-task model to a new additive distribution of binding values that is different from the distributions of the original sets (factor1: new binding value distribution). In addition, the model is usually more sensitive to the labeled values in the regression task than to those in the classification task (factor2: data type sensitivity). Furthermore, the number of classification interactions used herein (238,949) is more than ten times that of regression interactions (22,321) (factor3: more samples). These factors partially explain why the multi-task model achieves better performance on the classification task than on the regression task.

Second, as shown in Fig. 4, the datasets used in our study have different sparse distributions in the interaction space and thus lead to “negative transfer” on a specific dataset. Negative transfer refers to information learned from a source domain that has a detrimental effect on a target learner and results in performance degradation [25]. In addition, the six main datasets employed for the multi-task model may not be able to cover adequate portion of the protein–ligand interaction data space to improve model performance markedly. Instead, heterogeneity between datasets may introduce noise for specific tasks and challenge the generalizability of model applications. Therefore, we hypothesize that the fusion of data from similar datasets may lead to a positive transfer effect and improve the accuracy of the model, while the fusion of data from different datasets disadvantages the model. To prove this assumption, we use another independent set, a list of purchasable compounds by target. This dataset is closer to PDBbind in the interaction space after applying a dimensionality reduction than the other datasets used in this study (Additional file 1: Figure S2). As shown in the Additional file, the single-task model trained only on PDBbind achieves RMSE = 1.476 for this test set, whereas the multi-task model trained on all the main datasets obtains RMSE = 1.648 (Additional file 1: Figure S3).

### Biological interpretation

Although deep learning is fast and accurate, it is difficult to know why it performs well on some tasks. Hence, we introduce a method, namely occlusion, to explore how our model discerns biological data. As defined in the “Occlusion” section of the Methods, the range of K_ij_ is (0, +∞). K_ij_ value greater than 1 indicates the masked subsequences are important for binding prediction because the gap between the masked predicted value and real value increases. The higher K_ij_ value means the greater importance of the corresponding masked region. K_ij_ value equal to 1 indicates the masked region will not affect the prediction. In addition, K_ij_ value less than 1 indicates the masked subsequences maybe a noise that do not involve in the binding, because of the reduction of the prediction error.

**Fig. 5:**
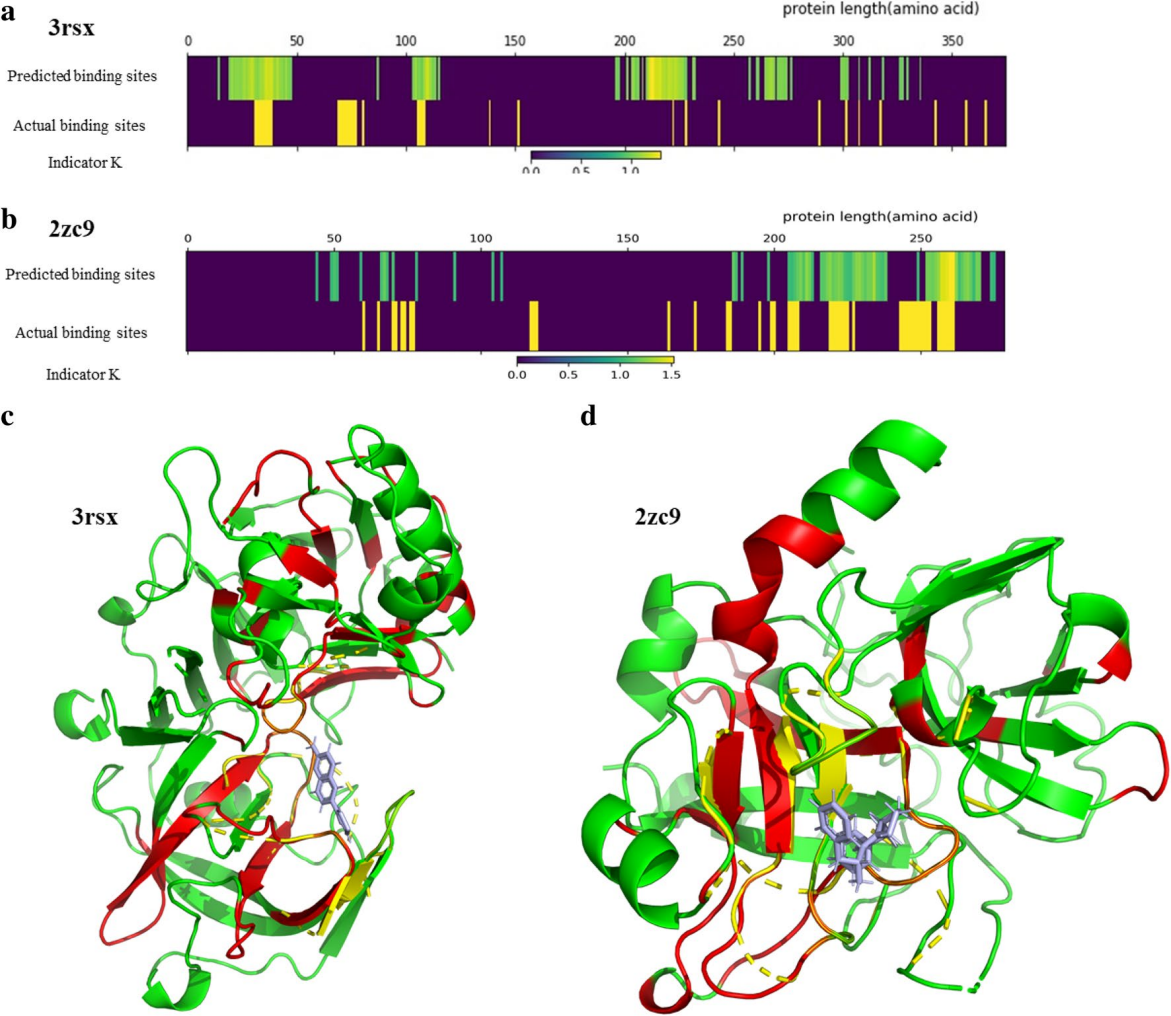
Alignment and visualization of the predicted and actual binding sites of protein sequences. Heat maps of the alignments between the predicted and actual binding sites: **a** 3rsx; **b** 2zc9 (the abscissa axis is the length of the protein sequence). Visualization: **c** 3rsx (the complex of Bace-1 (beta-secretase) and inhibitor 6-(thiophen-3-yl) quinolin-2-amine);  2zc9 (the complex of thrombin and inhibitor d-phenylalanyl-*N*-(3-chlorobenzyl)-l-prolinamide). The basic protein structures are present in green. The predicted important sites, which are highlighted in red, nearly overlap with the actual binding pockets (yellow) and cover the protein residues that interact with the ligands (light blue)

Heat maps of two examples are illustrated in Fig. 5a, b. The yellow regions within the actual binding sites (bottom) indicate the binding pocket for ligands. For the predicted binding sites (upper), the color of the regions are more close to yellow means more important to the binding prediction by our model. Clearly, the predicted binding sites, especially high-K regions, are very close to the ground truth, suggesting that our deep learning model indeed processes the data correctly. It should be noted that the local translation invariance caused by CNN pooling may result in a slight shift in the alignment. Besides, the center point selection of the masked region may also affect the shift. Understanding these phenomena is meaningful because we pay more attention on a specific region or domain, rather than single amino acid.

The 3D structures of the selected protein-ligand complexes are also visualized in Fig. 5c, d. Figure 5c shows the complex of Bace-1 (beta-secretase) and inhibitor 6-(thiophen-3-yl) quinolin-2-amine (PDB ID: 3rsx). Asp32, Tyr71, Phe108 and Asp228 are the key residues and form a pocket to interact with the inhibitor. These residues are detected correctly with high K values by our model. In the complex of thrombin and inhibitor d-phenylalanyl-*N*-(3-chlorobenzyl)-l-prolinamide, the key residues Asn98, Ile174, Glu217 and Lys224, which located close to the C-terminus of the target, are also detected, unlike the pocket located in the central part of 3rsx. These results indicate that our model is more likely to exploit key residues involved in the actual interaction regardless of their positions (six more examples are illustrated in Additional file 1: Figures S4–S6). However, some actual binding residues are not detected, and some false binding sites are highlighted, indicating that some important residues may impact the binding indirectly. It is also possible that allosteric sites within proteins may be part of this prediction. For example, ITK (interleukin-2-inducible T-cell kinase) has an ATP pocket and an allosteric pocket, which are in complex with 4-(carbamoylamino)-1-(7-propoxynaphthalen-1-yl)-1 H-pyrazole- 3-carboxamide (PDB ID: 4m13) and 4-(carbamoylamino)-1-(naphthalen-1-yl)-1 H-pyrazole-3- carboxamide (PDB ID: 4m0y), respectively. Our predicted results highlight most of both pockets while most nonbinding sites are not highlighted (Additional file 1: Figure S7).

Furthermore, we analyze the predicted important sites across the PDBbind samples. We set the parameter Kr (Kr∈[0,100]) to tune K_threshold_, and then calculate average precision and recall across the whole set according to Kr. The PR curves presented in Additional file 1:  Figure S8 indicate that our model performs better than the random guess approach in predicting binding sites. Combining the occlusion algorithm with our prediction is helpful for locating the binding pockets of known protein–ligand complexes and finding new and important binding regions of unknown complexes. It should be emphasized that our model uses only sequence information as input and that these results are achieved not only on the training dataset but also on the test dataset with unknown complexes.

## Conclusions

In this paper, we propose an interpretable multi-task deep learning model to predict the protein–ligand interactions. Previous studies either employed identical datasets or focused on a single task, hindering performance comparison across these models. Here, we introduce multiple task modules into the deep learning model, which can unify protein–ligand datasets with multiple labels. It should be noted that our model achieves competitive performance with 3D structure based deep learning models with the same training set size. Although the 3D complexes retain complete binding information, the amount of such data is excessively small, and an end-to-end model cannot extract a sufficient number of common features. The poor performance on unknown complexes may be a general problem for structure-based predictive models (i.e., poor generalizability). Additionally, our model outperforms all 20 state-of-the-art scoring functions on the CASF-2013 ‘scoring power’ benchmark.

Different databases may contain different data distributions, which challenge the applicability of predictive models. The proposed multi-task model unifies different tasks, allowing the model to use more data with various types of labels, thus performing fitting in a wider sample space. Although the multi-task model may result in small performance loss on individual datasets, especially during regression tasks, the model applicability is increased by unifying multiple datasets effectively.

The interpretability of deep learning model is a question worth investigating. Although they are high performance, deep learning models always suffer from “black box” issue and may yield good results because of misleading reasons. Interestingly, our model, together with the proposed occlusion algorithm, can be employed to pinpoint the critical amino acids for protein-ligand binding. We again emphasize that our model finds these binding sites with a relatively high accuracy by using only sequence information. We are enthusiastic about the future applications of such sequence-based multi-task predictive models. More datasets and more tasks involving drug toxicity and sensitivity prediction would be beneficial to improve the generalizability and applicability of our model.

## Materials and methods

### Data

We use ten datasets in this study, including six main datasets and four independent sets (Table 7). The main datasets consist of four classification sets (DUD-E, *Human*, *C. elegans* and KIBA) and two regression sets (PDBbind and Davis). The independent sets include two regression sets, CASF-2013 and Astex Diverse, and two classification set, MUV and BindingDB.

The Directory of Useful Decoys Enhanced (DUD-E), a benchmark dataset for evaluating structure based virtual screening methods, is used for classification [26]. The target clustering method is applied to avoid redundancy between the training and testing sets. Similar to Ragoza et al., 3-fold cross validation is used to evaluate our model, and proteins are clustered using global sequence alignment to ensure that targets with greater than 80% sequence identity are included in the same fold during cross-validation. The negative-to-positive ratio of is set to 3:1 to avoid an imbalance of data. A total of 91,220 samples from DUD-E dataset are used in this study. Additionally, the maximum unbiased validation (MUV) dataset, which consists of 17 targets, each with 30 actives and 15,000 decoys, is used as an independent test set. In this study, 9 of 17 targets with structural information are used to make comparisons with structure-based methods. Similarly, the *Human* with 3369 positive and 10,107 negative samples, and *C. elegans* datasets with 4,000 positive and 12,000 negative samples are used for classification [16]. The kinase inhibitor bioactivity (KIBA) dataset is used for classification [27]. Similar to a previous study [28], the KIBA threshold of 3.0 becomes 12.1 after transformation and protein–ligand interactions with values bigger than 12.1 are regarded as positive samples. All 118,253 samples from KIBA are only used for classification in this study due to the different bioactivity values within the dataset. A dataset containing 2706 positive and 2802 negative samples, which was carefully curated from BindingDB database, is used as an independent test set [24].

The PDBbind v.2016 database, which provides structural complexes with the corresponding binding affinity data (K_d_, K_i_), is used for regression [29]. To evaluate the generalizability of our model, the CASF-2013 benchmark with 195 complexes and the Astex Diverse set with 73 complexes (samples without binding affinity and those present in PDBbind (1YVF in the general set) are excluded) are used as additional independent test sets [21, 30]. Specifically, we split the PDBbind set exactly the same way as Pafnucy to facilitate a fair comparison. Briefly, the procedure is described as follows. (i) The whole core set (290 complexes) is used as an external test set. (ii) A total of 1000 complexes (same as Pafnucy) from the refined set are used for validation. (iii) The remaining complexes from the refined and general sets are used for training. Thus, 13,196 complexes from PDBbind are used for regression. Similarly, the kinase dataset Davis consisting of a total of 30,056 interactions with the corresponding binding affinity (K_d_) is used for regression [31]. It should be noted that most samples within Davis have binding values of 5, which would cause an imbalanced distribution for our total dataset. Thus these samples are removed and a total of 9,125 samples of Davis are used. Finally, 271,816 interactions are used in this study, including 22,589 for regression and 249,227 for classification.

**Table 7:** An overview of relevant datasets

Dataset	Type	# Proteins	# Interactions	# Positive to negative ratio
DUD-E*	Classification	102	91,220	1: 3
Human	Classification	2488	13,476	1:3
C. elegans	Classification	2496	16,000	1:3
KIBA	Classification	229	118,253	4.7:1
MUV*	Classification	9	4770	1:16
BindingDB	Classification	466	5508	1:1
PDBbind*	Regression	13,196	13,196	–
Davis	Regression	442	9125	–
CASF-2013*	Regression	195	195	–
Astex Diverse*	Regression	73	73	–

Asterisk (*) indicates structure dataset

Best values are higlhlighted in bold

### Model

#### Single‐task model

Our model contains two main parts, extracting features by convolutional layers to get protein/ligand embedding vectors and predicting their interaction by processing the concatenated vectors by fully connected layers (Fig. 1).

In more detail, we use one hot encoding to represent protein and ligand. The number of unique tokens of protein amino acid and ligand SMILES is 20 and 64, respectively. For each protein, its sequences are encoded and padded at the end to produce a 20 × 1200 matrix. Proteins with residues shorter than 1200 are padded to that length, whereas residues longer than 1200 are cut off to ensure that all inputs have the same size. Similarly, for each ligand, its SMILES identifiers are encoded and padded to produce a 64 × 200 matrix. Then, the two input matrices are processed by three CNN blocks. More specifically, each block consists of two convolutional layers and one pooling layer, Additionally, the inception block [32] is used instead of the VGG block [33] in the last two convolution blocks. The inception block consists of convolutional kernels with different sizes, including 1 × 1, 3 × 3, 5 × 5 and a 3 × 3 max pooling layer. After feature extraction, the protein and ligand are embedded to 1024 dimensional vectors and the two vectors are concatenated to feed into three dense layers, the units of which are 512, 64 and 1. A multi-dropout layer is added after each dense layer to reduce overfitting. Each multi-dropout layer consists of five units generating random dropout values, and then the final dropout is calculated by the weighted mean of these values to achieve better performance. We employ the rectified linear unit (ReLU), sigmoid function and linear function as activation function for middle layers, classification output layer and regression output layer, respectively. At last, the model generates five different values in the last dense layer and combines them into the final output.

#### Multi‐task model

Based on the architecture of the single-task model, the multi-task model contains two main parts: shared layers for learning general hidden features from all data and task-specific layers for learning specific weights for different tasks [34]. Here, we have two different tasks: binary classification and regression. The input, feature extraction and concatenation parts are similar to those of the single-task model. The loss functions for different tasks are defined as: binary cross-entropy for classification (Loss1) and the MSE with L2 regularization for regression (Loss2).

1 $$Loss1=\frac{1}{N}\sum _{i=1}^{N}{y}_{i}\text{log}\left(f\left({x}_{i};w\right)\right)+(1-{y}_{i}\left)\text{log}(1-f\left({x}_{i};w\right)\right)$$

2 $$Loss2=\frac{1}{M}\sum _{i=1}^{M}{(g\left({x}_{i};w\right)-{value}_{i})}^{2}+\lambda \| w \| ^{2}$$

where *N, M* and *f*(), and *g*() correspond to the samples and models for the classification and regression tasks; *x*_*i*_ and *y*_*i*_ correspond to the input and labels, respectively. ||*w*|| is an L2 regularization term; and *λ* ≥ 0 is used to adjust the relationship between the empirical risk and regularization term of the regression task.

### Training

#### Single model

We initialize the model weights using Glorot uniform initializer, which corrects the variance of uniform, keeping the variance of output and input from each layer the same [35]. We use the Adam optimizer to train our model, and set the initial learning rate and batch size to 10^− 4^ and 128, respectively. Besides, other default parameters are set similar to He et al. [36]. Then, we monitor the training process by early stopping and select the final model with the minimum loss on the validation set.

#### Multi‐task model

The input for a multi-task model is usually one sample with several labels corresponding to different supervised information. Different from the common situation, here we have samples with different single label (i.e., one sample one label), for classification or regression. Thus we design an alternate training method.

At beginning, the training sets containing classification and regression samples are randomly split into multiple batches. Then, each batch of the regression task and the classification task are alternately trained. In other words, alternate training is applied during which the back propagation algorithm selects different branches to update according to different sources of data. In each training batch of regression/classification task, the shared weights and the specific weights are updated. Similar to the training process of single-task model, we select the final models based on the minimum values on their corresponding validation sets.

### Evaluation

We use three measures including root mean square error (RMSE), standard deviation (SD) and Pearson’s correlation coefficient (R) to evaluate the performance of our model on the regression task. RMSE and Pearson’s correlation coefficient R are used to calculate the differences and the linear correlation, respectively, between the predicted and real values:

3 $$RMSE=\sqrt{\frac{1}{N}\sum _{i=1}^{N}{({y}_{i}-{\hat y}_{i})}^{2}}$$

4 $$R=\frac{\sum _{i=1}^{N}({y}_{i}-\stackrel{-}{y})({\hat y}_{i}-\stackrel{-}{\hat y})}{\sqrt{\sum _{i=1}^{N}{({y}_{i}-\stackrel{-}{y})}^{2}}\sqrt{\frac{1}{N}\sum _{i=1}^{N}{({\hat y}_{i}-\stackrel{-}{\hat y})}^{2}}}$$

5 $$SD=\sqrt{\frac{1}{N-1}\sum _{i=1}^{N}\left[{y}_{i}-{({a\hat y}_{i}+b)}^{2}\right]}$$

where N is the size of a dataset, *y*_*i*_ is the real value (experimentally measured binding affinity) whereas *ŷ*_*i*_ is the predicted value. $\stackrel{-}{\hat y}$ is the average of real values whereas $\stackrel{-}{y}$ is the average of the predicted value.

Various metrics including precision, recall, F1 score, specificity, AUC and MCC (Matthews correlation coefficient), are used to evaluate the classification performance. The formulas are listed below:

6 $$Accuracy=\frac{TP+TN}{P+N}$$

7 $$Precision= \frac{TP}{TP+FP}$$

8 $$Recall=\frac{TP}{TP+FN}$$

9 $$Specificity=\frac{TN}{FP+TN}$$

10 $$MCC=\frac{TP \times TN- FP \times FN}{\sqrt{(TP+FP)(TP+FN)(TN+FP)(TN+FN)}}$$

where TP is the number of true positives, TN is the number of true negatives, FP is the number of false positives, FN is the number of false negatives, P indicates positive, and N indicates negative.

### Occlusion

We present a nonparametric algorithm called “occlusion”, which was derived from the field of computer vision [37] and was simply introduced at our conference presentation [38], to explore which parts of the input sequences are critical to the task. Compared with the real binding pockets in proteins, we can measure the accuracy of this method. Basically, for a sample consisting of a protein input matrix and a ligand input matrix, we mask the protein matrix along sequence while keeping ligand matrix unchanged. Then, different masked regions of protein will lead to different output values and the important of each region can be quantified by comparing to the original predicted value.

Specifically speaking, subsequence mask is performed along the protein sequence direction, generating the corresponding occlusion result. The sliding windows size and the stride is set to 15 and 1, respectively, after numerous experiments. Then, the importance of the masked subsequence can be calculated by comparing the occlusion result and the original predicted value. An evaluation measure K is defined below to quantify the importance of masked regions:

11 $${K}_{ij}=\frac{\left|{p}_{ij}-{v}_{i}\right|}{\left|{p}_{i}-{v}_{i}\right|+\epsilon }$$

where *v*_*i*_, *p*_*i*_ and *p*_*ij*_ represent the real binding value, the predicted value of complete sample and the predicted value after occlusion, respectively. ε is a small positive real number to avoid the denominator equal to 0. After integrating all K values of one sample, the importance of each part of protein can be visualized.

## Supplementary Information

**Additional file 1.**

## Data Availability

All used data are available from the PDBbind/DUD-E/KIBA/DAVIS/Human/C. elegans databases. The original publications of these databases have been cited in our manuscript. The code and datasets are available at https://github.com/Siat-Code/Multi-PLI/.

## References

[1] Ma D-L, Chan DS-H, Leung C-H (2013). Drug repositioning by structure-based virtual screening. Chem Soc Rev.

[2] Koeppen H, Kriegl J, Lessel U et al (2011) Ligand-based virtual screening. virtual screen princ Challenges, pract Guide 61–85. 10.1002/9783527633326.ch3

[3] Varnek A, Baskin I (2012). Machine learning methods for property prediction in Chemoinformatics: Quo Vadis ?. J Chem Inf Model.

[4] Lo Y-C, Rensi SE, Torng W, Altman RB (2018). Machine learning in chemoinformatics and drug discovery. Drug Discov Today.

[5] Krizhevsky A, Sutskever I, Hinton GE (2017). ImageNet classification with deep convolutional neural networks. Commun ACM.

[6] Voulodimos A, Doulamis N, Doulamis A, Protopapadakis E (2018). Deep learning for computer vision: a brief review. Comput Intell Neurosci.

[7] Young T, Hazarika D, Poria S, Cambria E (2018). Recent trends in deep learning based natural language processing. IEEE Comput Intell Mag.

[8] Chen H, Engkvist O, Wang Y (2018). The rise of deep learning in drug discovery. Drug Discov Today.

[9] Wallach I, Dzamba M, Heifets A (2015). AtomNet: A Deep Convolutional Neural Network for Bioactivity Prediction in Structure-based Drug Discovery. Data Min Knowl Discov.

[10] Ragoza M, Hochuli J, Idrobo E (2017). Protein-Ligand Scoring with Convolutional Neural Networks. J Chem Inf Model.

[11] Stepniewska-Dziubinska MM, Zielenkiewicz P, Siedlecki P (2018). Development and evaluation of a deep learning model for protein–ligand binding affinity prediction. Bioinformatics.

[12] Öztürk H, Özgür A, Ozkirimli E (2018). DeepDTA: deep drug–target binding affinity prediction. Bioinformatics.

[13] Tsubaki M, Tomii K, Sese J (2018). Compound-protein interaction prediction with end-to-end learning of neural networks for graphs and sequences. Bioinformatics.

[14] Lee I, Keum J, Nam H (2019). DeepConv-DTI: Prediction of drug-target interactions via deep learning with convolution on protein sequences. PLOS Comput Biol.

[15] Wan F, Zeng J (2016). Deep learning with feature embedding for compound-protein interaction prediction. bioRxiv.

[16] Liu H, Sun J, Guan J (2015). Improving compound-protein interaction prediction by building up highly credible negative samples. Bioinformatics.

[17] Sieg J, Flachsenberg F, Rarey M (2019). In need of bias control: evaluating chemical data for machine learning in structure-based virtual screening. J Chem Inf Model.

[18] Chen L, Cruz A, Ramsey S (2019). Hidden bias in the DUD-E dataset leads to misleading performance of deep learning in structure-based virtual screening. PLoS ONE.

[19] Fu L, Niu B, Zhu Z (2012). CD-HIT: accelerated for clustering the next-generation sequencing data. Bioinformatics.

[20] Karpov P, Godin G, Tetko IV (2020). Transformer-CNN: Swiss knife for QSAR modeling and interpretation. J Cheminform.

[21] Li Y, Han L, Liu Z, Wang R (2014). Comparative Assessment of Scoring Functions on an Updated Benchmark: 2. Evaluation Methods and General Results. J Chem Inf Model.

[22] Trott O, Olson AJ (2009). AutoDock Vina: Improving the speed and accuracy of docking with a new scoring function, efficient optimization, and multithreading. J Comput Chem NA-NA.

[23] Wu Z, Ramsundar B, Feinberg EN (2018). MoleculeNet: A benchmark for molecular machine learning. Chem Sci.

[24] Yingkai Gao K, Fokoue A, Luo H et al (2018) Interpretable drug target prediction using deep neural representation. IJCAI 2018:3371–3377

[25] Weiss K, Khoshgoftaar TM, Wang D (2016). A survey of transfer learning. J Big Data.

[26] Mysinger MM, Carchia M, Irwin JJ, Shoichet BK (2012). Directory of useful decoys, enhanced (DUD-E): Better ligands and decoys for better benchmarking. J Med Chem.

[27] Tang J, Szwajda A, Shakyawar S (2014). Making sense of large-scale kinase inhibitor bioactivity data sets: A comparative and integrative analysis. J Chem Inf Model.

[28] Heidemeyer M, Cherkasov A, Ester M (2017). SimBoost: a read-across approach for predicting drug–target binding affinities using gradient boosting machines. J Cheminform.

[29] Wang R, Fang X, Lu Y, Wang S (2004). The PDBbind database: collection of binding affinities for protein–ligand complexes with known three-dimensional structures. J Med Chem.

[30] Hartshorn MJ, Verdonk ML, Chessari G (2007). Diverse, high-quality test set for the validation of protein-ligand docking performance. J Med Chem.

[31] Davis MI, Hunt JP, Herrgard S (2011). Comprehensive analysis of kinase inhibitor selectivity. Nat Biotechnol.

[32] Szegedy C, Vanhoucke V, Ioffe S et al (2015) Rethinking the inception architecture for computer vision.

[33] Simonyan K, Zisserman A (2014) Very deep convolutional networks for large-scale image recognition.

[34] Jiang J, Hu F, Zhu M, Yin P (2019) A multi-task deep model for protein-ligand interaction prediction. In: 2019 International Conference on Intelligent Informatics and Sciences B (ICIIBMS). IEEE, pp 28–31

[35] Glorot X, Bengio Y (2010) Understanding the difficulty of training deep feedforward neural networks. In: Teh YW, Titterington M (eds) Proceedings of the Thirteenth International Conference on Artificial Intelligence and Statistics. JMLR Workshop and Conference Proceedings, Chia Laguna Resort, Sardinia, Italy, pp 249–256

[36] He K, Zhang X, Ren S, Sun J (2015) Delving Deep into Rectifiers: Surpassing Human-Level Performance on ImageNet Classification. In: 2015 IEEE International Conference on Computer Vision (ICCV). IEEE, pp 1026–1034

[37] Zeiler MD, Fergus R (2014) Visualizing and Understanding Convolutional Networks. In: European conference on computer vision (ECCV). pp 818–833

[38] Hu F, Jiang J, Yin P (2019) Interpretable Prediction of Protein-Ligand Interaction by Convolutional Neural Network. In: 2019 IEEE International Conference on Bioinformatics, Biomedicine (BIBM). IEEE, pp 656–659

