# Supplementary material for: Multi-PLI: interpretable multi‐task deep learning model for unifying protein–ligand interaction datasets
Source: J Cheminform. 2021 Apr 15;13:30. doi: 10.1186/s13321-021-00510-6 (PMC8051026; doi:10.1186/s13321-021-00510-6)
Supplement: Supplementary file 1 — Additional file 1. [file 13321_2021_510_MOESM1_ESM.docx]

# Additional file


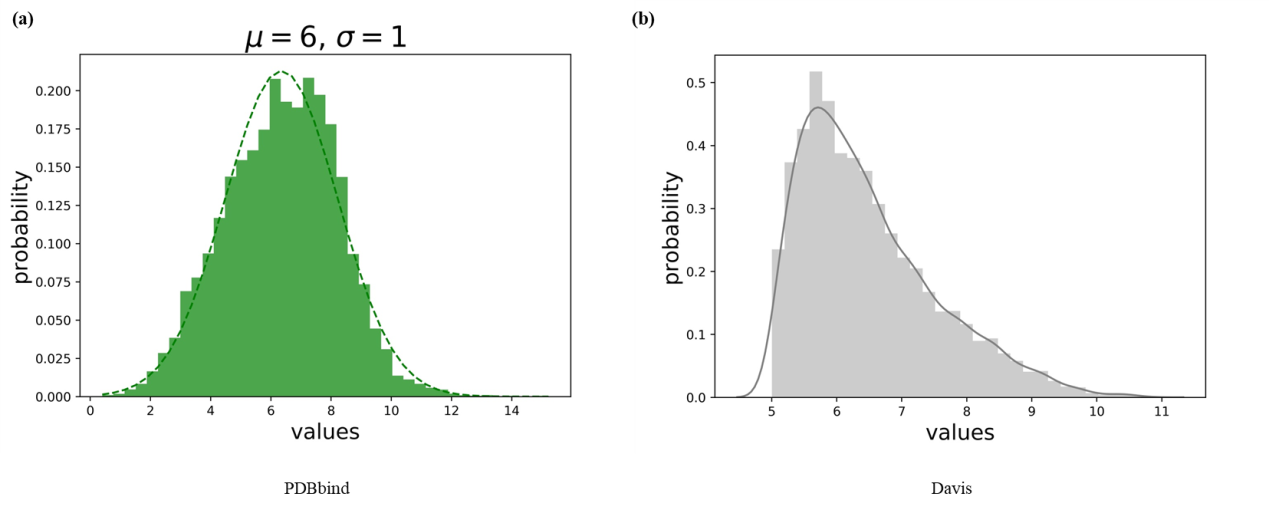


Supporting Figure S1. The interaction value distribution of PDBbind and Davis. (a) PDBbind set fits well to normal distribution whereas (b) Davis obeys a skew distribution which most distributes at 6.


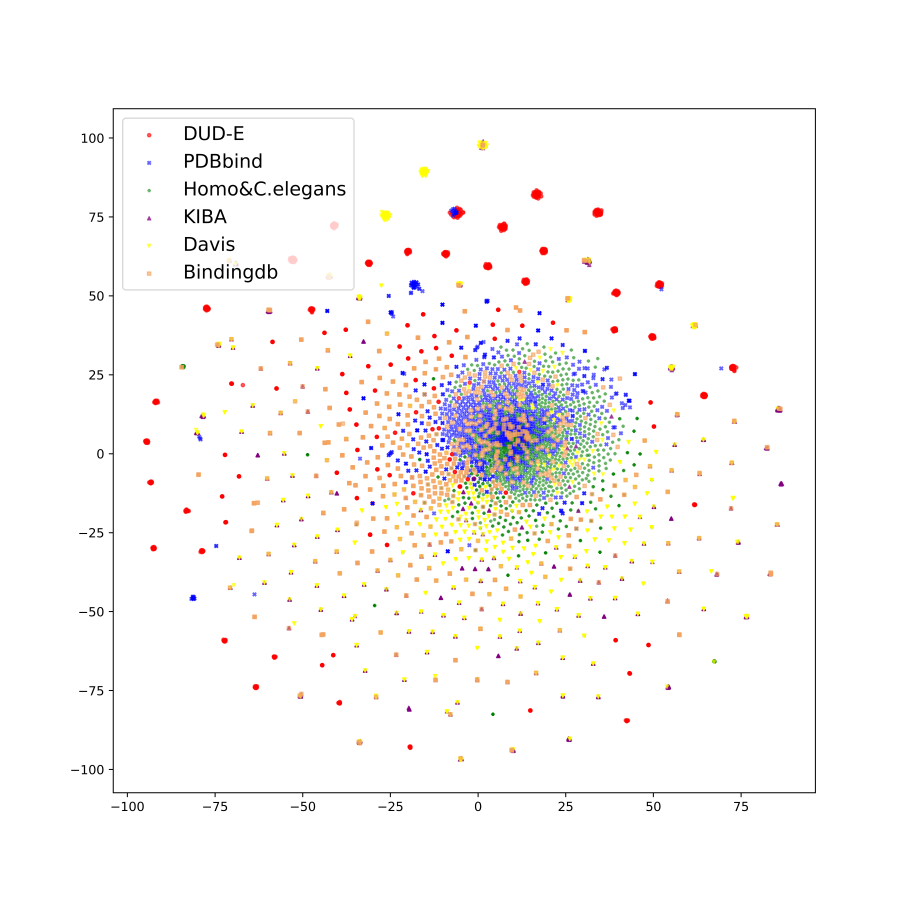


Supporting Figure S2. Visualization of datasets distribution by t-SNE

Each color indicates a protein-ligand dataset. The visualization result indicates that DB dataset is closer to PDBbind in the protein-ligand space after dimensionality reduction as compared to other datasets used in this study.


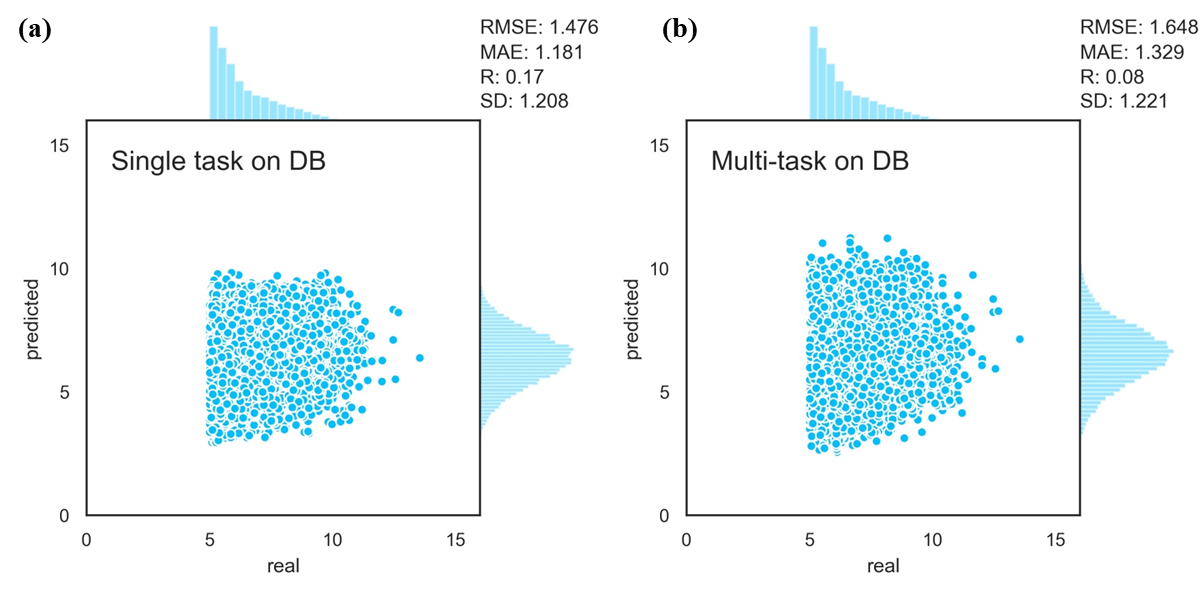


Supporting Figure S3. Predictions for Independent testset DB by single (left) and multi-task (right) models

The single task model trained only on the PDBbind achieves RMSE=1.476 for DB test set whereas multi-task model trained on all five datasets gains RMSE=1.648 for DB.


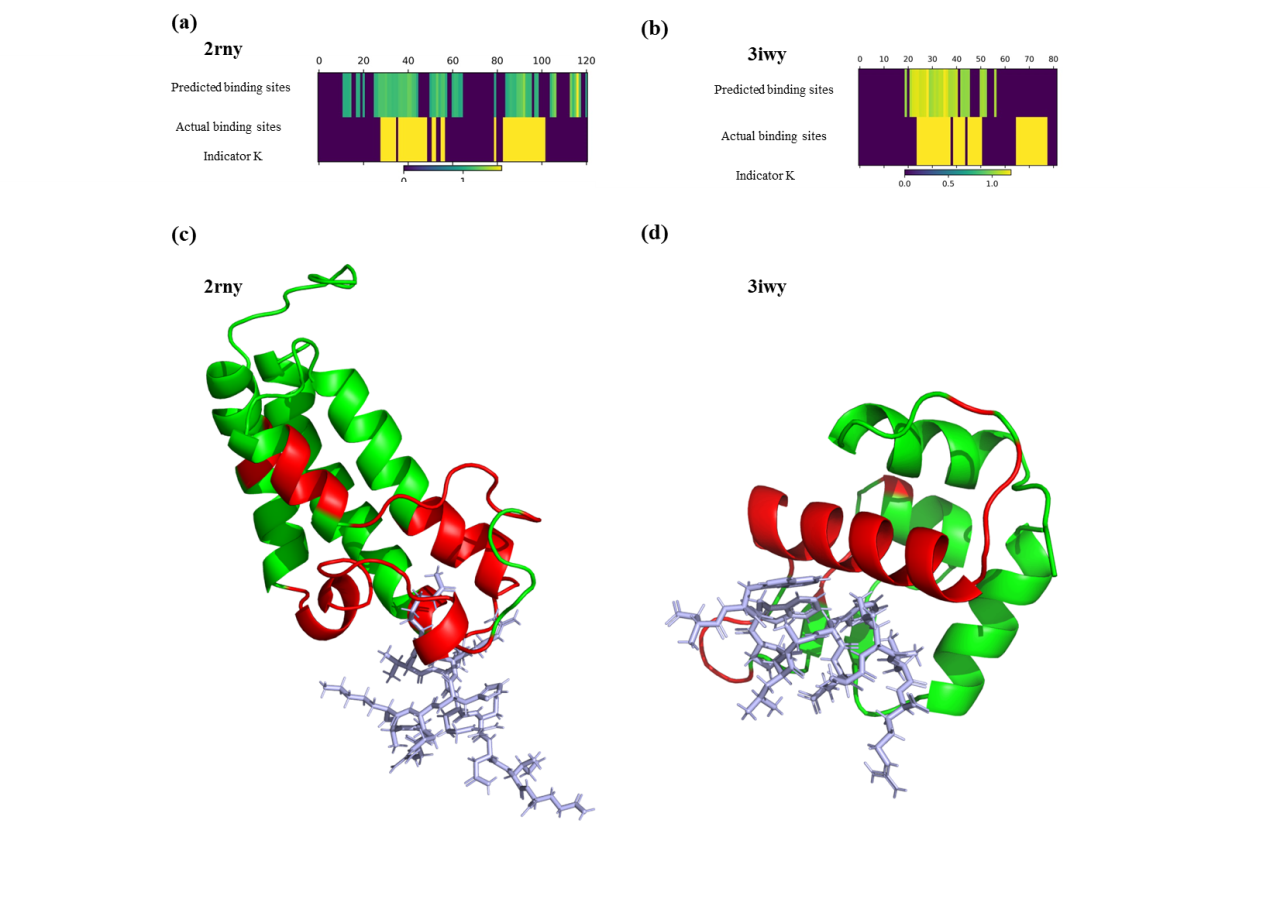


Supporting Figure S4. Visualization of predicted important sites and actual binding pocket. The heat map of alignments between predicted and actual binding sites: (a) 2rny; (b) 3iwy; Visualization: (c) 2rny; (d) 3iwy. The predicted important sites, which are highlighted by red, nearly overlap with actual binding pocket (yellow) and cover the protein residues that interact with ligands (white).


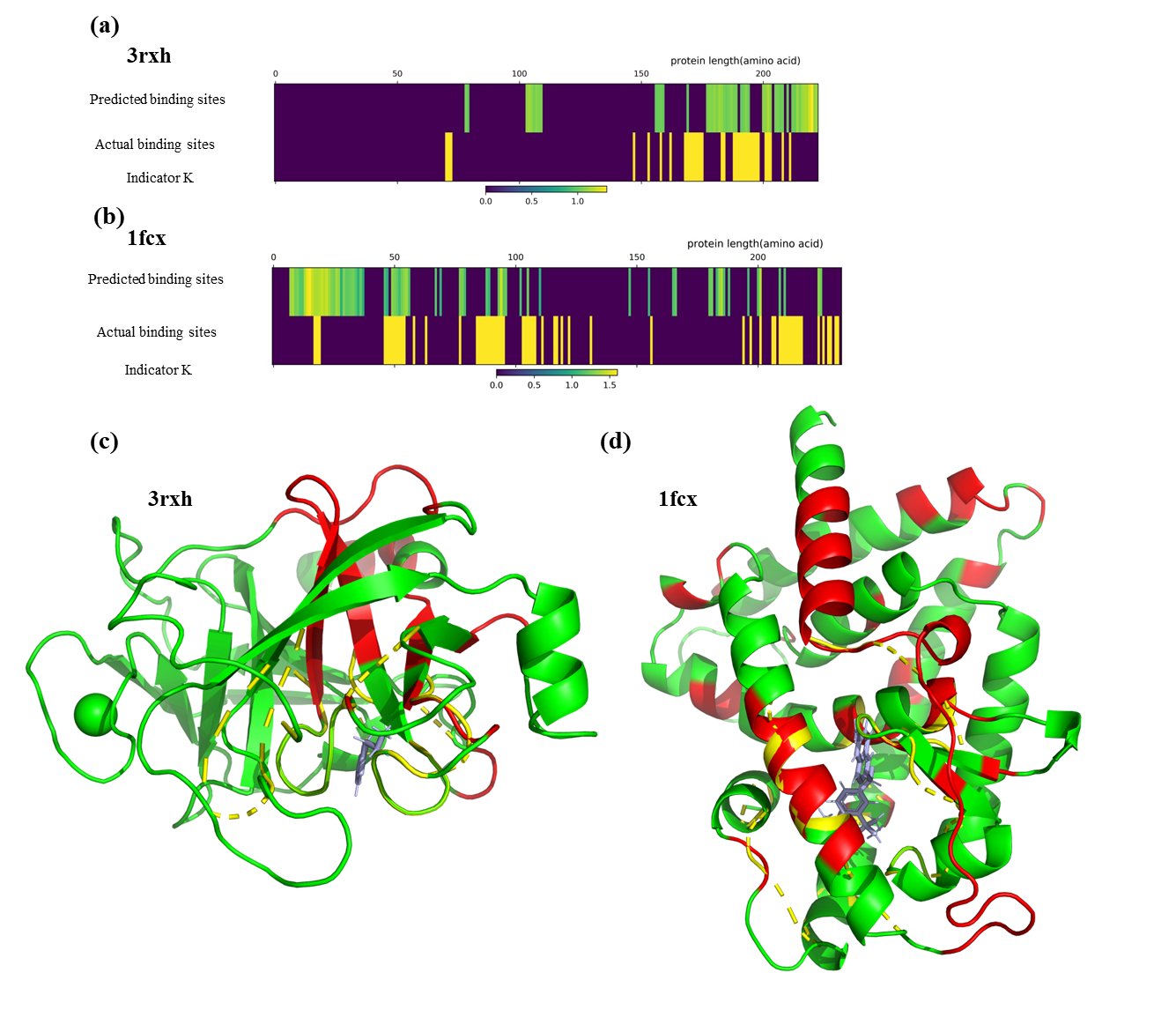


Supporting Figure S5. Visualization of predicted important sites and actual binding pocket. The heat map of alignments between predicted and actual binding sites: (a) 3rxh; (b) 1fxc; Visualization: (c) 3rxh; (d) 1fcx.


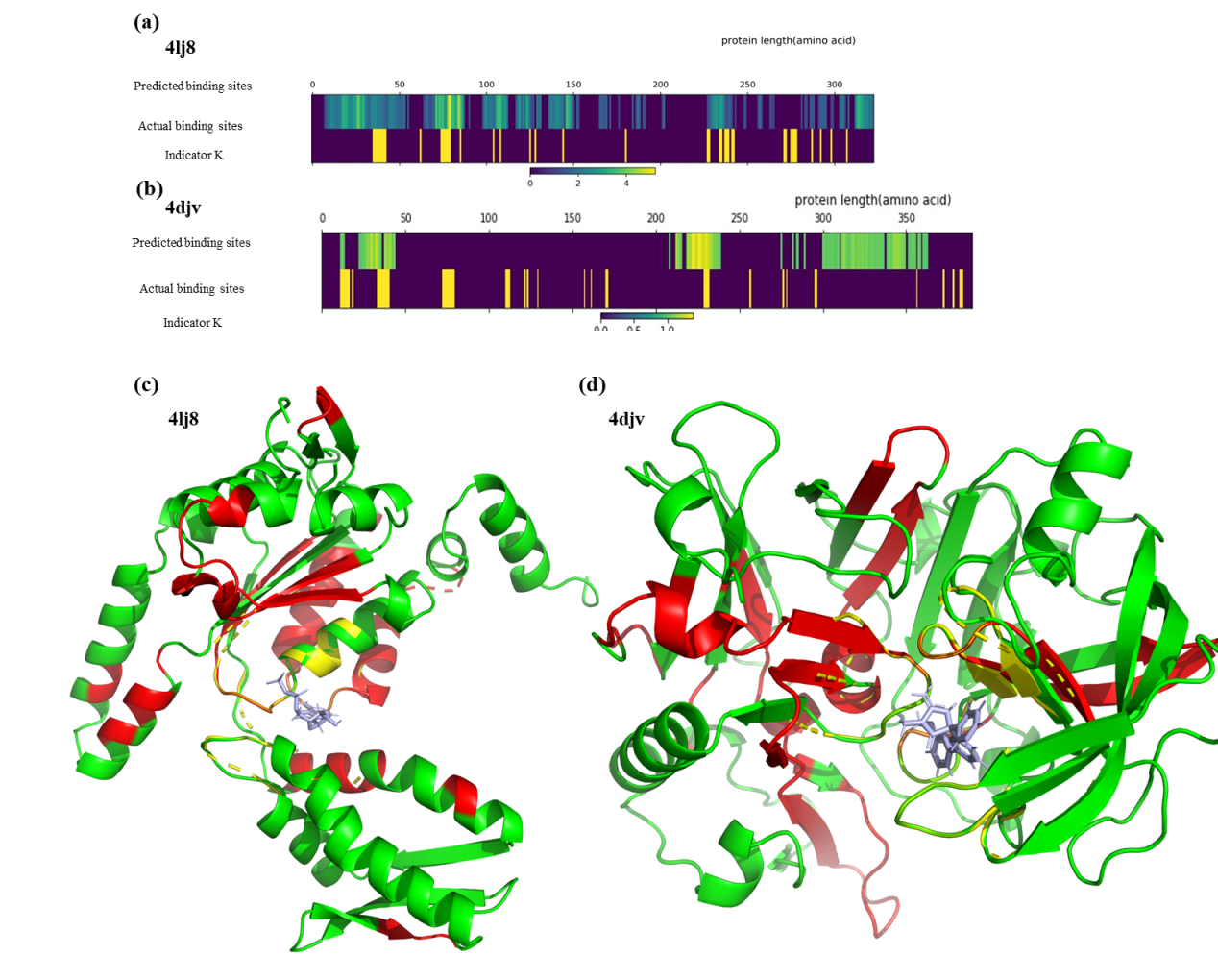


Supporting Figure S6. Visualization of predicted important sites and actual binding pocket. The heat map of alignments between predicted and actual binding sites: (a) 4lj8; (b) 4djv; Visualization: (c) 4lj8; (d) 4djv.


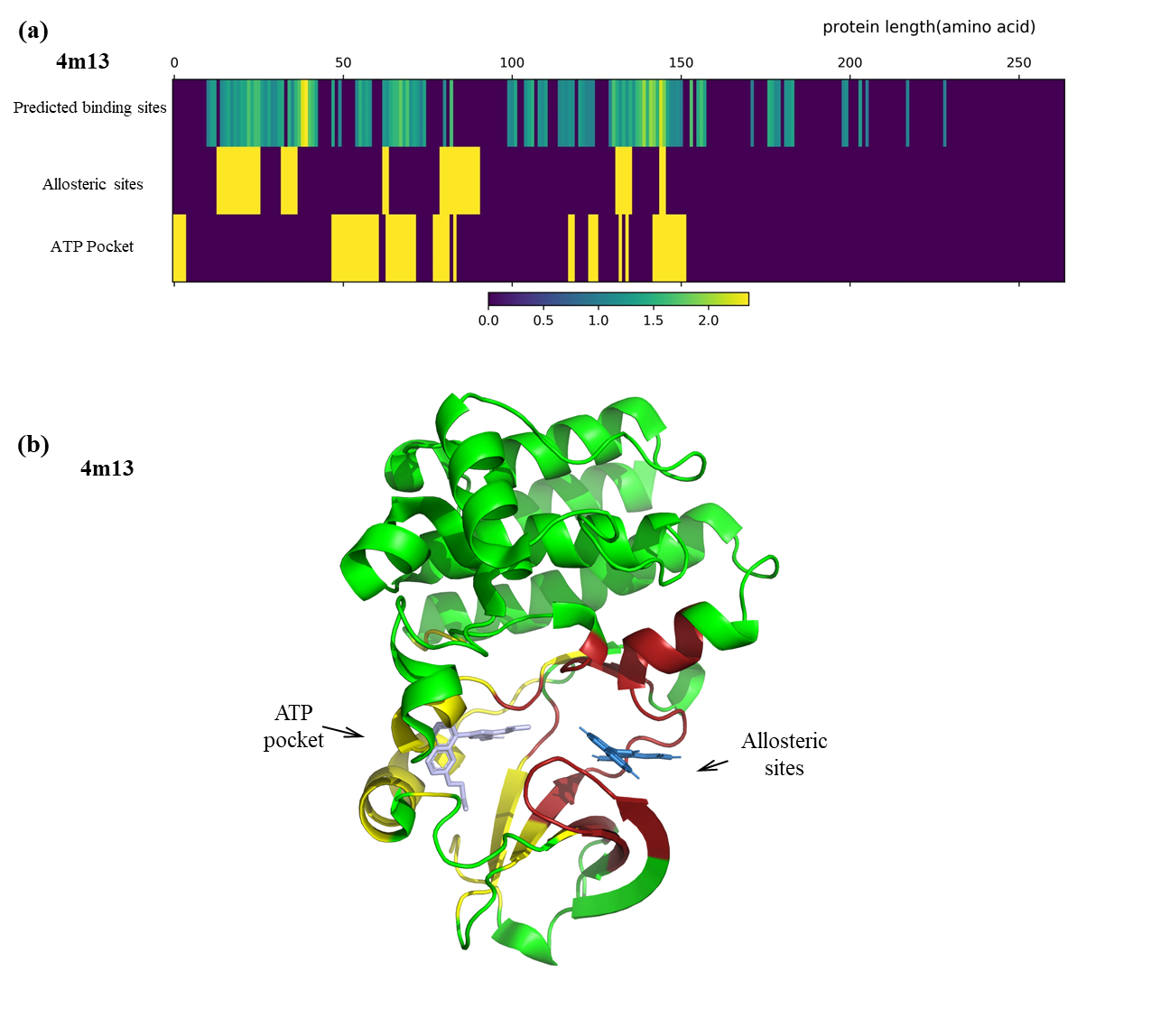


Supporting Figure S7. Visualization of predicted important sites, actual binding pocket and allosteric sites. (a) The heat map of alignments between predicted, allosteric sites and actual binding sites; (b) Crystal structures of ITK in complex with 4-(carbamoylamino)-1-(7-propoxynaphthalen-1-yl)-1H-pyrazole-3-carboxamide (ATP pocket, PDB ID: 4m13) and 4-(carbamoylamino)-1-(naphthalen-1-yl)-1H-pyrazole-3-carboxamide (Allosteric sites, PDB ID: 4m0y).


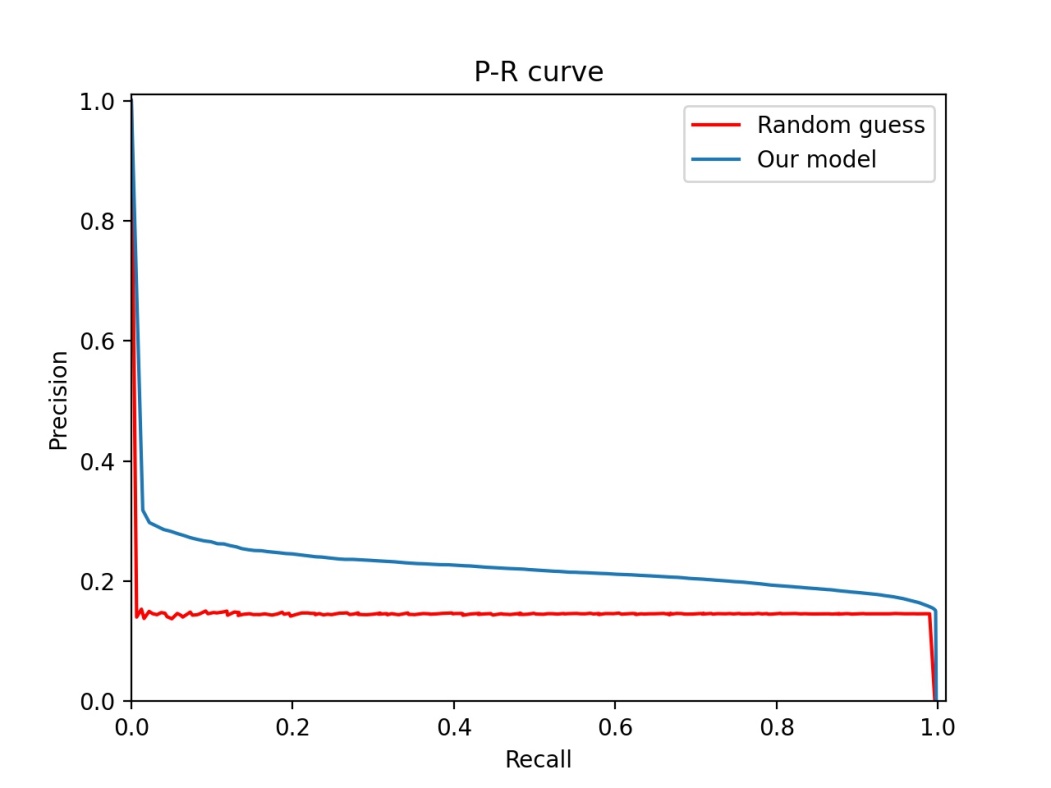


Supporting Figure S8. The PR curves of predicted binding sites by our model and random guess. For single protein (the length is n), it has n predicted K values (K∈[0, Kmax]), defined as vector A. In the loop of Kr, Kthreshold = numpy.percentile(A, Kr), and the predicted values in A that bigger than Kthreshold are conserved as important sites, and then the corresponding Precision and Recall could be calculated after alignment with actual binding sites. The average Precision and Recall across the whole set could be calculated according to Kr.


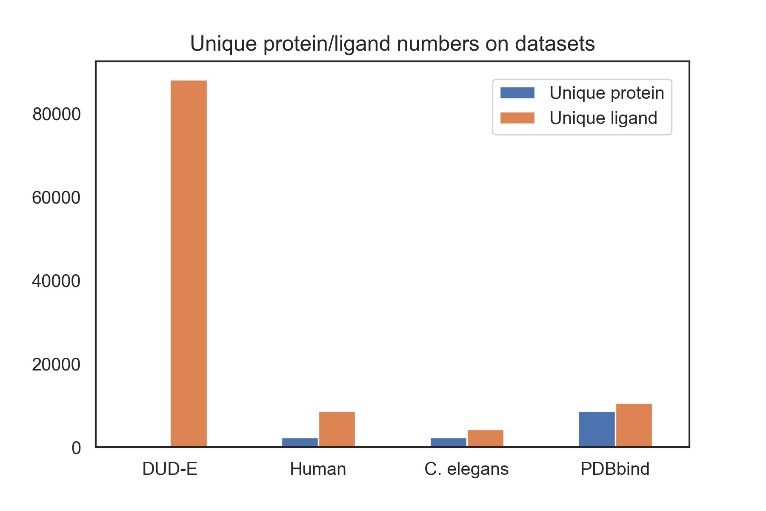


Supporting Figure S9. Unique protein/ligand numbers on several datasets.

Supporting Table 1. Ablation study on DUD-E, Human and C. elegans

| **Dataset** | **Ablation** | **acc** | **auc** | **precision** | **recall** | **F1** | **balanced acc** | **mcc** |
| --- | --- | --- | --- | --- | --- | --- | --- | --- |
| DUDE | complete | 0.909 | 0.959 | 0.853 | 0.773 | 0.810 | 0.864 | 0.753 |
|  | without protein | 0.906 | 0.956 | 0.837 | 0.776 | 0.805 | 0.863 | 0.745 |
|  | without ligand | 0.75 | 0.5 | 0 | 0 | 0 | 0.5 | 0 |
| Human | complete | 0.920 | 0.958 | 0.826 | 0.799 | 0.812 | 0.876 | 0.761 |
|  | without protein | 0.881 | 0.883 | 0.811 | 0.591 | 0.683 | 0.776 | 0.624 |
|  | without ligand | 0.858 | 0.855 | 0.812 | 0.447 | 0.577 | 0.709 | 0.532 |
| C.elegans | complete | 0.943 | 0.963 | 0.884 | 0.803 | 0.841 | 0.889 | 0.808 |
|  | without protein | 0.922 | 0.901 | 0.926 | 0.634 | 0.753 | 0.811 | 0.726 |
|  | without ligand | 0.873 | 0.873 | 0.703 | 0.556 | 0.621 | 0.751 | 0.551 |

Supporting Table 2. Ablation study on PDBbind

| **Dataset** | **Method** | **RMSE** | **R** | **SD** |
| --- | --- | --- | --- | --- |
| PDBbind | complete model | 1.44 | 0.76 | 1.43 |
|  | without protein | 1.51 | 0.68 | 1.50 |
|  | without ligand | 1.82 | 0.55 | 1.82 |

Supporting Table 3. Model performance on classification datasets

| **Dataset** | **tasks** | **acc** | **auc** | **precision** | **recall** | **F1** | **balanced acc** | **mcc** |
| --- | --- | --- | --- | --- | --- | --- | --- | --- |
| DUDE | single | 0.925 | 0.973 | 0.823 | 0.893 | 0.857 | 0.915 | 0.808 |
|  | multi | 0.923 | 0.971 | 0.872 | 0.809 | 0.839 | 0.885 | 0.789 |
| Human | single | 0.920 | 0.958 | 0.826 | 0.799 | 0.812 | 0.876 | 0.761 |
|  | multi | 0.930 | 0.961 | 0.840 | 0.837 | 0.838 | 0.896 | 0.794 |
| C.elegans | single | 0.943 | 0.963 | 0.884 | 0.803 | 0.841 | 0.889 | 0.808 |
|  | multi | 0.942 | 0.970 | 0.839 | 0.851 | 0.845 | 0.907 | 0.809 |

Supporting Table 4. The AUC scores of each target on MUV

| Target | Vina | 3D-CNN on DUD-E | Single-task (DUDE) | Multi-task |
| --- | --- | --- | --- | --- |
| 466 | 0.593 | **0.663** | 0.434 | 0.415 |
| 548 | 0.460 | **0.791** | 0.467 | 0.446 |
| 600 | **0.578** | 0.559 | 0.529 | 0.510 |
| 689 | **0.596** | 0.514 | 0.400 | 0.366 |
| 692 | 0.413 | **0.480** | 0.315 | 0.367 |
| 832 | **0.610** | 0.402 | 0.418 | 0.452 |
| 846 | **0.655** | 0.384 | 0.500 | 0.545 |
| 852 | **0.515** | 0.348 | 0.435 | 0.388 |
| 859 | 0.517 | 0.560 | 0.578 | 0.494 |
